# Discussions of Mississippi Valley Dental Association

**Published:** 1866-10

**Authors:** C. W. Spalding


### Proceedings of Societies.

### DISCUSSIONS OF MISSISSIPPI VALLEY DENTAL ASSOCIATION.

Held February 21, 22, and 23, 1866.

REPORTED BY DR. C. W. SPALDING.

*Dental Materia Medica.*

PROF. GEO. WATT, of Xenia, Ohio, opened the discussion. He stated that he had recently written on this subject, having commenced a series of articles for the "Dental Register," in which he proposed to treat of it at considerable length. In examining the subject let us first define the term Dental Materia Medica. We employ this term as denoting a specialty of medical science—a science which draws upon all the kingdoms of nature for substances capable of modifying the laws of life. Premising that there is but one system of medicine—that there is room for only one, as there is for but one God—it becomes our duty to try to discover and determine that system and apply it to our practice.

*Dental* materia medica would therefore have relation to those substances which exert an influence on diseases of the dental organs, and on those of the other organs and tissues of the mouth. In a legitimate sense, the scope is much wider than is generally supposed. Many fine operators who consider it their duty to treat all cases of disease originating in the mouth, would think they were going beyond the limits of their specialty in prescribing for diseases which do not *originate* in that cavity, although they may exist there. If the general practitioner prescribes for such cases, they belong none the less to dental surgery; and the dentist will probably find that he is not alone in being at a loss how to treat these cases successfully Young dentists should certainly prepare themselves to treat all such diseases. Ample opportunity is afforded them for obtaining a thorough dental education; and they should not be excused if they neglect to avail themselves of it.

There is no necessity for drawing so nice a line between dentistry and medicine as some do. Dentistry is a branch of medicine; and if the dentist knows what to do in any particular case just as well as the physician, I don't see why he should not do it, provided he all the time adheres to *dental* materia medica. I have not entered into the discussion of the *modus operandi* or specific action of any particular drug, but trust this will be done before this subject is disposed of. Some of us are more familiar with the action of one particular remedy, and others of another. I hope each one will speak of such as he is most familiar with.

Dr. McCullum, of Kentucky, said that his success in the treatment of diseased and receding gums had not been very satisfactory. He had used creosote and iodine, as recommended by Dr. W. A. Pease, of Dayton, Ohio.

Prof. J. Taft said that formerly manipulative dentistry was considered the important thing in dental practice. Filling teeth and making artificial substitutes had been looked upon as the all of dental art and science; while the knowledge of the causes and treatment of diseases was a matter that had been almost wholly neglected. A man who confines himself wholly to the mere manipulative arts of dentistry is not much elevated above a watch-maker, or other skillful mechanic. He was happy, however, to bear testimony to the existence of an advanced state of professional attainment among the better class of practitioners. With this class, the best means of preventing, and the most successful methods of treating disease were now considered of the very first importance.

Such minds were constantly on the stretch to obtain information on these important points. They study to discover how the dental organs can best be preserved and maintained in a state of complete and perfect health. They seek to prevent the occurrence of disease rather than to confine themselves to combatting it after it has become established. The practitioner of medicine often fails to recognize the sympathy existing between teeth that are diseased and certain systemic conditions; he makes little or no effort to arrest the progress of decay or to prevent absorption of the gums. These examples are mentioned to show that the physician does not regard these things as properly belonging to his department. They therefore must receive the attention and care of the dentist.

Now, medicines are either local or general in their action; and the dentist should thoroughly understand the nature and effects of every remedy he employs. He should clearly see the diseased condition which he proposes to change or modify, should perceive how it may be changed, and what remedy will best produce the desired effect. All this should be clear in his mind, otherwise it is empiricism.

Dental materia medica is a subject that is not yet well understood. Some know but little, while few know all about it. Still, every one should so well understand the action of the remedies in more common use as to know what effects they will produce in any given case. Let us take, for example, tannin, or creosote. A few years ago, a knowledge of the therapeutic action of these drugs was considered almost unattainable. Now they are well understood, even by students. Comparisons like this serve as landmarks to show the progress we are making.

Dr. W. W. Allport, of Chicago—We hear a great deal said about giving remedies to produce certain effects; but we have no illustrations of the manner in which these remedies act. What is the *modus operandi*, the therapeutic law according to which any given remedy produces its effects? We know the cause, and we can see its effects; but we cannot trace the connection between them. Why, for instance, will creosote and iodine cause the restoration of the gums and the alveolar processes? Should like to hear this matter discussed.

Dr. Taft—Has endeavored to ascertain facts and draw legitimate deductions, and in this way arrive at a proper conclusion on this subject. It is always desirable before introducing therapeutic treatment to ascertain as nearly as possible the true condition, and also the cause of that condition. There is an infinite variety of pathological states; these are modified by previous physiological conditions, and by different causes.

All are aware of the fact that in the absence of vigorous physiological action, exciting causes produce greater results. Oftentimes apparently very slight local causes will produce great disturbance. Sometimes a totally foreign body will remain in the tissues with impunity; while in others the same substance will produce the most serious results. In some cases even a vitiated secretion serves as an active irritant. There is not only a very great diversity in the manner in which different constitutions will tolerate the presence of offensive agents, but almost if not quite as great a difference in the ability to dispose of or remove such agents. In one case a little spiculæ of bone will occasion severe and extensive disease; while in another case, sentinels will seem to be placed about it that will so hedge it in that it is perfectly harmless; while in still other cases it is speedily dissolved and removed.

All these things and many others should be well understood before coming to a full decision as to what should be done in the way of treatment.

The ordinary diseases of the teeth are well understood, both in nature and cause, by the thorough dental student. The local causes are more readily apprehended than the remote, and may be understood to some extent where there is but circumscribed knowledge. But to take in and fully understand the nature and influence of remote causes, involves the widest range of physiological and pathological knowledge.

In treating diseased tissue, organ, or body, the first thing in order to be accomplished is to remove or neutralize the injurious agent. This is sometimes done by mechanical means—a surgical operation; or by medicinal agency, by the administration of those things that will attack the enemy directly, or will rouse the powers of nature to such a point that the foe will be literally driven out.

The principles here hinted at must be well understood before we can intelligently apply therapeutic agents. We may understand most fully the nature of the agents, but unless its action on living tissue is understood, the administration will be empirical and uncertain.

Dr. Allport asks, What would you give to correct the constitutional condition which causes the secretions to become acrid, and *why* would you give it?

Dr. Watt replies: Would give iodide of potassium, and would use it because it is a really good thing. It is composed of one equivalent of iodine and one of potassium. These substances have affinities for each other, but they also easily separate in obedience to other affinities, and enter into combination with other substances. They would not readily reach the capillaries separately, because they have such strong affinities for other substances which they would encounter on the way. But in combination they help each other along (the compound being nearly inert locally) until the capillaries are reached, when they are separated, and each is then left free to form new combinations, and to exert its own chemical powers—potassa, by dissolving these abnormal tissues, or rather the materials of which they are composed; while iodine, having affinity for other substances, decomposes certain tissues, by taking away one of the parts of which they are composed, and thus changes their character. This latter, also, by its stimulating properties, increases the action of the absorbents. All medicines act chemically, and in that way only. The body is itself a chemical laboratory. All eliminations and excretions are conducted on chemical principles. Suppose a case of tertiary syphilis.

This is a disease of the bone-forming process itself. We know that bones are all the time changing—constantly dying and being renewed. Neither syphilis nor scrofula change the condition of bone material already formed—they affect the manner in which the process of renewal is carried on. Potassium unites with oxygen and forms potassa. This solvent holds other deleterious substances in solution, till they are passed off through the kidneys and by perspiration. Thus is brought about what we call an alterative action on the system.

In syphilis, we will suppose that the tertiary state is not yet reached, but the processes are wasting away. Here iodide of potassium is just as applicable as in the former case, readily correcting the abnormal action of the tissues, and thus arresting the progress of the disease. To illustrate: Chlorine acts on gold only when in contact with it in its nascent condition. So potassa and iodine, resulting from the decomposition of iodide of potassium, their nascent condition, combines with these substances or poisons, and they are carried out of the system, while in this state of solution, by means of the excretory fluids.

The local action of iodine and creosote, when applied to the gums and alveolar processes, is that of an escharotic. This is also a chemical process; these substances uniting, by chemical affinity, with the poisoned tissues, a slough is formed, when nature sets up increased action below in her efforts to effect a restoration.

In answer to a question, Dr. Watt stated that he preferred small doses to large ones; usually prescribed three grains of iodide of potassium three times a day, increasing the dose, if necessary, to ten grains. The first indications of the constitutional effects of this remedy are pallor of the face and a slight disturbance of the appetite. Soon after the appearance of these symptoms, some improvement was generally observed.

Dr. Allport remarked that he had derived more information from this discussion than he had ever before received on any one subject in the same length of time.

Adjourned till to-morrow.

---

*Remaining discussions reported by* Dr. C. M. Wright.—*Ed.*

---

It was with great reluctance that I complied with the request of the society in making the attempt to report the doings and sayings of members, at this meeting; and it is with greater unwillingness of mind that I sit down to the task of arranging my imperfect notes for the printer and the "reading dentist." My dislike is not because I am not as anxious as any, that the many wise and useful remarks, the interesting and instructive discussions of the society should be recorded and made common property, but that necessarily so much must be lost, and all the balance so imperfectly reported by my inexperienced hands. Like a wrecker, however, when a noble ship has been dashed to pieces on the shore, I have made a desperate effort to save a few of the valuables; and though they will be presented in a somewhat damaged condition, I hope they will be acceptable. If, in the following report, any member is made to appear dull or uninteresting, or if I have put anything in his mouth with my pen, that he thinks his tongue never uttered; or again, if he has been very imperfectly reported, or entirely neglected, when he expected much more,—if he fails to make the brilliant *hit* in this report, that he thinks he did in his speech, that member is at perfect liberty to cry out against the reporter—to accuse him of stupidity, ignorance, or inability to perform what he has attempted, but not of malice aforethought. If this is not satisfactory—if the wound be not so easily healed—the aggrieved member may lay the charge at the doors of those who nominated and insisted that I should take the reporter's pen. If I may be allowed to suggest, the very best way of correcting all mistakes, making clear all dullness, sharpening and bringing out in all their original brilliancy the good points that do not appear in this report, would be, for the member to take up the pen himself, and arrange and present his remarks and opinions, in the form of an essay or *paper*, to the publisher of the *Register*. By thus doing, he would clear the conscience of the reporter of a great responsibility, and, at the same time, contribute to one of the most important and popular means of professional education, viz: Dental Periodical Literature.—*Reporter*.

*Thursday*, *P. M.*, *Feb.* 22, 1866.

Before commencing the discussion of the appointed subject, the society unanimously requested Dr. McKellops, of St. Louis, to exhibit some models and specimens which he had brought from Europe, as a present from Dr. Preterre, of Paris, to the Ohio Dental College. Dr. McKellops kindly consented, and exhibited a number of handsome and correct models of cases of congenital cleft palate, and other malformations of the mouth and adjacent parts, and the ingenious appliances to obviate the difficulties. Each case was described, and clearly explained by Dr. McK ; and the eagerness of all the members to see and hear, expressed forcibly the interest with which they regard this most important and interesting subject. An ingenious piece of mechanism for correcting irregularities of the teeth was also exhibited and explained. It consisted of a vulcanized rubber plate with metallic attachments, by which the teeth could be moved in almost every way desired. Dr. McK. also exhibited a number of root forceps, of shapes new and suggestive to Americans. We regret exceedingly that we cannot, in our report bring Dr. McKellops, and the models he exhibited bodily before you, and detain him here until all could taste the fruits he gathered during his late tour in Europe ; as he is in no way selfish about them, but gives freely what has evidently cost him much labor and study, not to mention money. The appreciation and thanks of the society can be but feebly expressed in words, though resolutions to this effect were passed. If all would imitate the generosity toward his professional brothers that Dr. McK. has shown, such rebukes as the gentleman gave to the course of a distinguished American Dentist, would not often be necessary. We are sorry we cannot report the remarks of Dr. McKellops, but without the models to illustrate, it would be unsatisfactory.

Dr. Harroun, of Toledo, explained his ingenious method of obtaining the impressions of mouths for the construction of artificial palatine obturators. The method can be better demonstrated than described in a report; and those who were not fortunate enough to be present must content themselves for the present, with the blissful state of ignorance *of the "proceedings of this society"* on the subject of cleft palates.

The subject of Materia Medica was again introduced for discussion as it had not been near exhausted in the morning.

Dr. J. Taft said he would like to hear the opinions of members about the modus operandi of the mercurials. The knowledge of the mode of action of mercury, in its various forms does not seem satisfactory in our own or even the Medical profession. The opinions extant on this subject appear crude and undeveloped, and he should like to have them made clearer and more generally understood. He wanted to know the whys and wherefores of the use of the mercurials, and how—*exactly* how, they act in the human system. Does, for instance, iodide of mercury, act in any way like iodide of potassium, or, as it was explained and described this morning. If it does not, he wanted to know just how it does act. He considered this as important a matter as any to our profession, and believed we could not be more profitably or practicably employed than in investigating such subjects.

Dr. Watt does not think iodide of mercury acts like iodide of potassium. Does not know that he can explain exactly how it does act. Sometimes the properties of the blood are inclined to be too adhesive, as in Pneumonia, or other diseases where there is a condition of mucus engorgement, and a tendency of the blood to fill up the tissues as by adhesion. When the blood is in this condition, this adhesive quality can be overcome by mercurials. Mercury is, perhaps, not favorable to the formation of new material in the system. In regard to the local action of mercury, he does not believe the popular idea to be true, that the teeth are corroded by its use—that it has a direct deleterious action on the teeth. Frequently after mercury has been used, the system may be in what might be called an acid condition. This condition is affected by the circumstances of the disease, of climate, of temperament, etc.; but the secretions about the teeth are acid, and the acid acts upon the teeth. But sometimes following the use of mercury, an alkaline condition is present, and the alkaline secretions affect injuriously the gums and mucous membrane. These conditions of the secretions follow the use of mercury, modified by many circumstances; and the teeth are as variously affected. The mercury itself is not the *direct* agent that causes the decay of the teeth.

Dr. Spalding was glad the action of medicines upon the teeth had been mentioned. He once believed that medicines would not affect the condition of the teeth after they were perfectly developed; but a few years ago he had a patient—a young lady—whose teeth were of good character, firm and strong. This lady had a spell of sickness—a lingering illness. Immediately after her recovery he examined her teeth and was surprised to find them friable and brittle, and changed entirely in quality of texture. Both the enamel and dentine were affected, and presented the same friable condition. With but little exertion, he could chip away the tooth with an excavator. He declined to operate for her at that time, and prescribed an acid, for medicine. As it was strawberry season, he told her to eat them freely, using sugar, believing that the acid of the Strawberry would accomplish the result; and it would be an agreeable way of taking medicine. After a few months, he again examined her teeth, and found them restored to their original good texture. Since that time he has believed that medicines, by certain chemical properties and changes, do have effect upon the character of the teeth, even after they are perfectly formed in adult mouths. In their systemic action, the character of the secretions are changed, and, by being taken into the circulation and uniting with certain properties or qualities of the blood, ar thrown out into the tissues. This patient had taken potassa, till the stomach had rejected it. The teeth had been changed by the alkaline action. The acid of the strawberry restored them. He had, therefore, based his theory upon fact, and wished to know upon what safer hypothesis he might build.

Dr. Cheesbrough had had a case very similar to the one mentioned by Dr. Spalding, and would like to know the *rationale* of the acid in strawberries, in such cases.

Dr. Spalding could not, in the case mentioned, tell exactly the *rationale*—he could only state the phenomena, and the facts; but in the operations of medicines in the system, there is another and a higher principle involved than the merely chemical. It is the dynamic—the vital principle. Chemistry cannot account for all the operations of medicine. A medicine does not act on a dead body as it does on the living. Life is required to account for the action of medicines in many cases. Vitality is as important in the operations of medicines as chemical affinities. To the dynamic principles as well as to the chemical, are we indebted for the curative properties of medicine. Chemical affinity is owing, in part, to this life principle, and can be accounted for in no other way. Mercury, for instance, seems to have a specific action—a tendency toward the osseous structure of the body, and is it not on account of an affinity of life qualities? A medicine may lose its power, or its life, and become a dead medicine. It may act then, perhaps, as the ghost of the medicine, on the ghost of a lost or dead member of the body. (?) Does not wish to insist upon the latter idea.—[Laughter.]

Dr. Allport cannot agree with the deductions of Dr. S., in regard to the case mentioned, of the young lady who ate strawberries. Thinks it probable that the alkaline administered injured the teeth, by affecting the animal portions of the bone, but should think the administration of acid would only complete the ruin, by breaking down and destroying the mineral portion of the teeth.

Dr. Watt said he did not understand a man when he talked about the dynamic forces of medicines. He believes only in the doctrine of chemical action. If the spirit—and he wished to be understood that he was as "spirituous" as any one need be—acts on the body, it must employ chemical means to do it. The devil himself, can seduce man only by causing chemical changes to take place in the human system; and only by the aid of chemistry can he do anything with man. In regard to the case mentioned by Dr. S., he said, any wasting disease may and does cause the conditions of the teeth described. In these diseases the nutritive functions do not work properly—they do not supply the quantity, or quality, necessary for the proper support of the body, or of the teeth. In consumption, in low typhus fevers, and in other debilitating diseases, the condition of the blood is changed, and all the bones in common with the rest of the body, fail because not properly nourished. He seldom operates for persons after a long and debilitating sickness has produced this diseased brittle condition of the teeth, till the *nutritive functions* have had time to correct the evil. When an alkaline condition of the blood and secretions is present, an acid given acts chemically, by neutralizing the alkalies in the blood, &c. To correct an acid state of the secretions, etc., which can easily be discovered by the use of litmus paper, in the mouth, from the saliva, or by the perspiration, he prescribes acetate of potash in doses of two or three grains.

Some discussion ensued here between Drs. Watt and Spalding in regard to the systems of medicine. Dr. Watt insisted that there was room for but *one system* of medicine, as there is for but one God in the universe. If there is room for two or more systems of medicine, then there may be room for two or more Gods, which he could not believe. For the science of Medicine has human nature as its basis, and claims all created things as its implements.

Dr. Spalding declared that while we, as scientific men, are searching for truth, and while medicine like every other science, has not arrived at a perfect state, and our knowledge of it is not infallible, we can not, with justice, claim any one as the only system, nor can we claim but one—the system of medicine. When we are perfect in our knowledge, and all consent that a conclusion has been arrived at, there may be but one system; but such is not the case to-day; nor is it likely to be for some time to come. While men of equal knowledge, equal scientific attainments, equal powers of judgement, differ on a question, it is not always the wisest plan to assert that there is room for but our, or one of the opinions.

Some further discussion on the same subject followed, Dr. McCullum said he did not expect to settle, forever, the agitated question, in the minds of all. He did not believe that, when he sat down, all would agree, and that nothing hereafter could be said on this subject of the chemical and dynamic forces, but he wished to say, clearly and conscisely, what he meant by these forces: By the chemical, the medicine acts on the system; by the dynamic, the system acts on the medicine.

Dr. Ulery here begged leave to introduce Mr. Hull, the inventor of a new blow-pipe. The machine was exhibited. It consists of a reservoir of japanned tin, which was filled about one third full of benzine, and into which air was forced by a pump. The generated gas was ignited at the end of a tube, about six or eight inches from the reservoir, and connected with it near the top. The flame could be regulated, and the heat from this would cook, roast, etc. Proceeding from this in a straight line, a small blow-pipe was attached; and quite a furious blast was forced out, which would solder for the dentist, when a small alcoholic flame was held near its mouth. The force of the blast could be regulated by screws or cocks. The society seemed very much interested in the machine; and Dr. Ulery and Mr. Hull were thanked for bringing it before them.

*Thursday Evening, Feb.* 22.

Quite an interesting, and even exciting debate on the mode of operation of arsenious acid in the teeth, occurred during the early part of this evening's session, which, we are sorry to say, must be lost to the readers of the *Register*, unless some member present at the time will present a report of it. Your reporter was unavoidably absent; but with your consent will take up the pen at the moment he entered the room, and begin the discussion with

Dr. Morgan, who in speaking of arsenic in dental practice, said he had learned from his experience, that in young persons, when the blood circulates rapidly the arsenic operates much more readily and rapidly. In many such cases, but thirty minutes are required to destroy the vitality of the pulp. In adults, also, where the pulse beats rapidly, a much shorter time is necessary for this medicine to accomplish its object than is usually prescribed.

Dr. H. A. Smith applies arsenious acid to devitalize the pulp, and generally requests his patients to return in from eight to twenty-four hours, but he has never had trouble with a case when the application, owing to the carelessness of the patient, has remained for several weeks in the tooth. There is certainly a very great variety of opinions in the profession, in regard to a medicine so universally employed as arsenious acid. The *authorities*, as well as those who do not presume to be authorities, differ widely in regard to the *modus ope-*

*randi*, and the time necessary for the complete devitalization of the pulps.

Dr. Berry, in his early practice used to apply arsenic to obtund the sensibility of dentine when the pulp was not exposed, but believes that, in forty-nine of fifty cases, where this practice is pursued in young patients of strumous habit, the pulp is devitalized.

Dr. Talbert has, for some time, used a paste made of arsenic and morphia, in about equal parts, and ground well together. Thinks the narcotic prevents or deadens the pain that so frequently follows the application of the mineral agent. Occasionally he used a very small quantity of this paste to destroy sensibility in the dentine. In this case, he applies it but for a very short time. Thinks that different cases require different quantities for the destruction of the life of the pulps. Temperament, texture of the teeth, &c., are circumstances which modify the action of this medicine. Stated a case where the alveolar process was affected by the arsenical application in the tooth.

Dr. Shadoan spoke of the porosity of tooth bone, which may be the cause of its sensibility in many instances, and insisted that arsenious acid—and all that class of compounds—is taken up by absorption, and produce devitalization of the pulp, even when a layer of dentine intervenes. He believes in the theory of the absorption of arsenic by the tubuli of the dentine, and asked if periostitis was not caused by arsenic being absorbed.

Dr. Taft agreed that the arsenious acid being absorbed by the pulp, &c., is the cause of the periostitis that so frequently follows the application. Authorities may differ on these points, they are at liberty to do so; but he contended that we are as capable of judging aright, and forming correct conclusions as any, on the operations of this agent. Temperament, and other conditions or circumstances, affect the action of this, as of almost every other medicine. Replied to a question that dead tissue, like a piece of wood or sponge, can absorb, and that there is no necessity for a tooth to be possessed of vitality to allow the transmission of arsenic.

Dr. H. A. Smith denies that there is any circulation in dead matter, and declines believing the correctness of the theory that arsenic is carried through the tooth, either by capillary or any other attraction. Does not regard it essential to subscribe to the doctrine of the absorption of arsenic, by the pulp or tubuli of the tooth, to account for the periostitis that frequently follows the destruction of the life in the pulp cavity. The devitalized pulp itself might cause an irritation which would produce periostitis. The destruction of blood vessels might so affect the circulation that, for a time, an abnormal condition would be present, and periostitis result. He could see no imperative necessity for hastily adopting the doctrine of absorption.

Dr. Watt. The arsenic combines chemically with the albuminous matter of the dentine; and an arsenate is formed, which fills up, or causes to be filled, the tubuli of the dentine. Decomposition takes place, layer or stratum at a time, perhaps, and this decomposition may proceed for months, even after the cavity has been duly filled. Death of the pulp may then result. Periostitis may occur, however, from the destruction of the contents of the pulp cavity from any cause. The normal circulation of blood is destroyed, and until nature has had time to provide a way to correct or regulate the results of the obstruction, periostitis may be the consequence. The artery that carries blood to the apex of the root, has been cut off, and irritation is produced for the time. He intends to try to clear his mind entirely of all he ever heard or thought he knew on this subject, and commence from the beginning to study, and to investigate it himself, and hopes that his attempt may be successful.

*Friday Morning Session.*

Dr. Berry believed that it would be more profitable to discontinue the discussion on Materia Medica, and also to omit the second subject in the order of discussions, and moved that "Filling Teeth" be accepted as the subject this morning. The society agreed, and the third subject offered by the executive committee was presented.

### Filling Teeth—Methods, and Materials Used.

Dr. J. Taft desired especially to hear from Drs. Morgan and Redman, being convinced that they were first class operators, and never having heard their methods described,

Dr. Morgan, uses soft foil in a majority of cases. Believes that any operator can make a plug hard enough to bear mastication with soft foil. He sometimes uses adhesive foil, and crystal gold, but considers the soft foil best in many cases. It can be more easily and perfectly adapted to the inequalities of the walls of cavities. He uses the file freely when necessary, and wood or rubber wedges. Very seldom uses instruments with serrated points. Sometimes in molar teeth, in making what he calls sort of two-story fillings, uses serrated points, after plugging the cavity or making the foundation with smooth points. Believes it a mechanical impossibility to make a perfectly solid filling with serrated points, on account of the fine indentations made by this kind of an instrument. Uses gold, and nothing but gold, in a large majority of fillings, and never fills a tooth with wet foil. Occasionally uses the mallet to condense his plugs. Though he does not discard entirely adhesive foil, he does not believe in its universal use.

Dr. Redman uses serrated points, and Abbey's foil No. 8. Commences his fillings by introducing as large cylinders as possible, and then filling up with smaller ones. Uses adhesive foil in some cases, but almost entirely fills with Abbey's soft foil.

Dr. J. Taft said there had been a great deal of disscussion of this subject of filling teeth, of the manipulation of gold, etc., and still it was not exhausted, and much could be learned. Spoke of the modifying circumstances which affected the result of the operation. The form of the instruments, the quality and condition of the gold, mood of the operator, physical and mental state of the patient, etc. All these circumstances should be viewed by the operator, and controlled if possible. The instruments should be in the best condition, and the *method* employed the one that brought the best results to the operator. The operator himself should regard the laws of health, and keep himself in a condition as perfect as possible, for the delicate work he has to perform. Would take him a long time to make good filling if he employed Dr. Morgan's method. He would have to train himself for work for a long time, though Dr. Morgan's results were good. It makes but little difference what method a man pursues, provided he attains the object—perfect fillings—with ease to himself and patients. Spoke of the occasional feeling of repugnance for certain patients that operators have—of an incompatibility between patient and operator, and considered it his duty not to operate for a person when this feeling existed, as this was certainly a condition that would affect the quality of the operations.

Dr. Morgan asked Dr. Taft if he had not had patients toward whom he had no feelings of repugnance, and yet who exhausted him exceedingly when he operated for them.

Dr. Taft said he had had such persons in his chair; and frequently where teeth are difficult to fill—where the walls of cavities are thin, and where there is constant danger from an overflow of the saliva, the operator's attention and energies are so strained that he becomes exhausted in a short time, and frequently breaks down or makes miserable fillings.

Dr. McClellan has experienced these feelings of exhaustion when he could not account for them by feelings of dislike, or the conditions of circumstances of the cases presented for filling. Thought it might be explained perhaps by the principle of endosmose or exosmose. Is it not similar to the phenomena, and may there not be a current of nerve force or of vitality established between the operator and patient? He believes we cannot do justice to this class of patients, and thinks it best to decline to operate for them. Where patients are very troublesome—always wanting to have their mouths examined, and where they occupy the time and wear the patience of the dentists, thinks we ought to charge them. Believes that if we charged for examinations we would not be troubled so much with a certain class of patients.

Dr. Berry suggested that the cause of the unusual fatigue at time might be found in the conditions of the atmosphere, owing to its electrical state, etc. Has experienced these feelings when he could account for them in no other way. Considers the health of the mouth a very important matter, if we wish to secure good operations. If the secretions are not in a healthy state, and if other conditions of disease are present, the permanency and perfectness of our work is affected; and we should not operate till the health of the parts surrounding the teeth is restored.

Dr. Sedgewick thinks that in many instances where nervousness, feelings of exhaustion and dislike to operate exists in the dentist, it is his own fault. Dissipation, the use of tobacco, and carelessness in regard to the observance of the laws of hygiene, are frequently causes of the inability to operate well. In his practice he uses nothing but gold and tin foils. His favorite gold foil is Watt's crystal, No. 4 or 6, which he anneals and manipulates with serrated points. Never has succeeded well with smooth pointed instruments. Has no fear of, and uses the file and the wedge. Does not confine himself to one method of filling. He uses the mallet or hand pressure, as he thinks best at the time.

Dr. Morgan hoped that some ready writer would prepare a paper for dentists, stating how, and in what condition they should keep themselves in regard to health, to operate with the best success. Afterwards he was the mover in an offer of a prize of one hundred dollars for the best essay on the subject. The motion will be found in the minutes of the proceedings.

Mr. Berry wished to question the propriety of the sentiment that many of the present day maintained, that we are so much superior to the old operators. Some of those who labored thirty, and even fifty years ago, used to fill teeth as thoroughly and well, and with as beautiful results as any dentist of the present. Believed that the operations of Hudson and Badger are not excelled by any of our first class operators. There were miserable operators in days gone by as there are now; but scientific men of the past were not so far behind us in their operations, as some are vain enough to think.

Dr. C. H. Harroun said, in referring to instruments for plugging, that in most of those sold at depots, the points were too sharp, or rather too long, the serrates were too deep. He prefers making his own points, which he does with a watchmaker's file.

Dr. Morgan repeated that the use of serrated points was a violation of mechanical principles.

Dr. Wells asked how a filling would be made of gold in a cavity situated in the posterior lingual and approximate angle of a lower molar, and of such shape that gold introduced at one side would pass through the cavity with no opportunity of stopping?

Dr. Sedgewick described an operation on a similar case, where the pulp cavity was filled, and an anchorage secured there.

Dr. McClellan described a method of filling, where only the lingual and labial walls of the tooth remained. A metal band is fitted perfectly to the tooth making two artificial walls, and reducing the cavity to a simple crown cavity.

Dr. Harroun, in such cases, fills one side first with amalgam, and then makes with gold a solid approximate filling. Afterwards, he removes the Amalgam, and replaces with gold by making another approximate filling. The Amalgam makes the walls for one filling, which in turn makes the wall for the other, after the amalgam is removed.

Dr. KcMellops, in cavities presenting this aspect, uses cylinders, which he rolls on a fine broach, making them (when the bottom of the cavity is smooth) of the length of the cavity. Laying this in the cavity, he compresses it firmly, making a foundation, which is the most important part of any filling. When the bottom of the cavity is uneven and deeper at the edges, he makes cylinders that taper to suit the inequalities, and placing them in the cavity, with their tapered ends together, condenses and gets his foundation. After a solid foundation is secured, other cylinders or blocks can be used, till the tooth is built up, and the operation finished. In preparing cavities, uses chisels, and in difficult approximate cavities uses a spade, or spoon shaped, and very small chisel, with spring tempered handle, and diamond point. He uses the wedge, and where practicable and necessary, rubber around the teeth. Places bibulous paper over the mouths of salivary ducts, to avoid overflows of saliva. He explained Dr. Coffin's method of filling by reflection. Dr. C. prepares his cavities with chisels, and cuts away the lingual wall in the incisors. With reflectors, magnifying mirrors, he fills from inside, standing upright at the chair.

Dr. James Taylor's method of filling that class of teeth where but two walls—the labial and lingual—remain similar to Dr. McKellops. He makes blocks that pass through the cavity at its base, and large enough to be retained themselves in the cavity, thus making a good foundation.

Dr. McClellan described his method of filling: In preparing for filling uses chisels and excavators of but two or three shapes. The hatchet shape is perhaps the most common. Uses napkins without stint, and seldom permits his fingers to touch the naked mucous membrane. Has abandoned the use of drills for making retaining points. Uses adhesive and soft gold foil, crystal gold, tin foil, and Wood's alloy, and favors the mallet, believing that better fillings are made with it. He then exhibited a plaster model of a mouth in which a number of teeth had been built up. Their crowns restored to their proper length, &c. In this case he had excavated around the pulps, making a kind of ditch to retain the gold; and in all cases of the kind endeavors to make his fillings something like a double headed rivet, with one head secured in the tooth, and the other out of it. In restoring crowns, uses crystal gold, unless where there is danger of moisture, when he uses crystal gold foil. Has filled with Wood's Alloy, and plated with gold in large cavities, which can be done by laying gold foil over the plug, and carefully passing the instrument, heated to the right temperature over it. In these cases he has never heard of any trouble from galvanic action.

Dr. Poore, Lexington, makes his own points to pluggers. Objects to their being very sharp; and, after serrating, polishes them to prevent their dragging the gold. Believes that they work better if they are made round at the edges. He uses the mallet, and especially in shallow cavities after having made good retaining points.

Dr. Shadoan said that when he had a very delicate operation of filling to perform, always wants the assistance of his mallet. He makes his retaining points either as grooves or pits. In incisors, when the walls are thin, and the gold is likely to show through the enamel, he uses good stiff white paper, believing that it assists the thin labial wall to resist the pressure of consolidation, and gives a white and more presentable color to the tooth than the glare of gold.

The society adjourned to meet at two o'clock, P. M.

*Friday Afternoon Session.*

Before the subject for discussion was introduced, Dr. H. A. Smith read a valuable and interesting paper on the Minor Operations of Dentistry, which was requested for publication by the society. It elicited many complimentary and confirmatory remarks from the members.

Dr. H. A. Smith asked for some light on the operation of bleaching devitalized teeth. He has used chloride of zinc, and many other preparations, with but comparatively poor success. Should like to know of a specific, and wished to hear the opinions, and of the practice of those who had been successful bleachers.

Dr. Morgan has had satisfactory results from the application of Chloride of Zinc. He has applied it frequently. Makes his applications every two or three days, and has observed the bleaching process going on even after the tooth has been filled.

Dr. Watt said in regard to Chloride of Zinc, that it decomposes slowly. The chlorine is the bleaching agent, and being liberated by the decomposition, effects the object, if time enough be allowed. Has noticed frequently that dentists have applied creosote before their bleaching agents, and wonder why they are not successful in restoring the lost color of the tooth. They seal up, with the creosote, the tubuli of the dentine, so that the bleaching process is retarded, or prevented entirely. Creosote should never be used before trying to bleach a tooth. He prefers the hypo-chlorites for bleaching agents ; for with them we obtain the bleaching properties of both chlorine and oxygen. In the chlorides (which decomposes more slowly too) we have but one active agent. The hypo chlorite of either soda or lime, is good for this purpose. Neither tannin nor creosote should be applied to the cavity when bleaching is to be done. Applies the hypo chlorite two or three times in an hour.

Dr. Talbert here read a paper, which was listened to with interest by all, on "Extracting Teeth," which in fact opened the subject for discussion for this session. In the essay read reference was made to the best position for the operator in extracting left superior molars. The author recommending, on account of the better control gained over the patient, and the easier application of the force required by the operator, the dentist to take his position on the left side of the chair, as nearly in front of the patient as possible.

Dr. Jas. Taylor agreed with the author of the essay in this particular, and, by request, demonstrated his position clearly, by taking a pair of forceps and assuming the exact attitude, on the rostrum, before one of the members. He stands on the left side of his operating chair, and nearly in front of the patient, holding between his thumb and fingers of his left hand, the alveolus about the left superior molar. Believes that in difficult cases of extracting these teeth, much can be gained by this position. He seldom uses the gum lancet.

Dr. H. R. Smith thinks the operator loses control of the patient by taking the position advocated by Drs. Talbert and Taylor. He prefers standing at the right of the operating chair, having support from it, and controlling perfectly the head of the patient. He disagrees with the author of the essay, in regard to cutting the gums before extracting. In many cases the gums are torn, and unnecessarily lacerated by using the beaks of the forceps in the place of a gum lancet. He regards the gum lancet a very useful instrument, especially before extracting adult's teeth.

Dr. Taylor favors the plan of forcing the forceps deep into the socket, believing that the root of the tooth is thereby forced out.

Dr. Shadoan prefers to take his position on the right side, in extracting any tooth, and considers this the true and scientific position for the dentist.

Dr. Watt. Some allusion having been made to the position of the fulcrum of the turn-key, insisted that the proper place for this relic of *barbarism* was the wall,—the key hanging by a nail. He prefers having the beaks of his forceps made long, so that any tooth can be grasped, and all necessity for using the turnkey be avoided.

Dr. Taylor in some isolated cases employs the turnkey with advantage. He would not like to be without one, though never uses it when anything else will do.

Dr. Poore, of Lexington, takes position on the right side of his chair, and has no delicacy about "hugging up" the heads of his patients. But very seldom uses the gum lancet. Believes in driving his forceps home when he is about to extract a tooth. Thinks the operator should *possess* himself. Is very successful in extracting teeth, and a large number of persons in his neighborhood have given him credit for extracting with but little pain.

Dr. Berry thinks that no operation in the province of our profession assists more in giving reputation to a dentist than extracting teeth if it is performed skillfully. Considers it a most important operation, and one that needs not only careful judgement and great caution, but knowledge and skill to perform it when necessary and in a proper manner. He has given much attention and earnest study to this branch of dental surgery for many years; yet when he hears of the great success, and the few failures of so many of his juniors in experience who seem to regard the operation so lightly, he feels a strong inclination to give up extracting teeth entirely.

Dr. McClellan said that the longer he practices, the more cautious he becomes in extracting teeth. Believes that a great many of the accidents attending this operation are the results of carelessness on the part of the dentist. He uses a small, delicately shaped, and sharp gum lancet, and passes it around and between the teeth, believing it important to preserve the septa of gum.

Dr. Morgan dislikes extracting exceedingly. Believes that it is an operation that carries a reproach to the profession with it. Thinks that too much care can not be bestowed upon the operation, that judgment and carefulness should precede, accompany and follow every performance of this business. Every tooth extracted is an evidence of inability to save the natural organ.

Dr. Sedgewick never extracts a tooth unless it *must* be removed. Seldom uses the gum lancet. Agrees with the gentlemen who believe in careful operations, etc.

The society adjourned to meet in the evening, at 7 o'clock.

### *Friday Evening.*

Before proceeding with the regular subject of discussion, Dr. Cushing read an exceedingly interesting essay on "Dental Education." He presented instructive and original views and valuable opinions on the various branches of this important subject. The essay was appreciated by the society and a copy requested for publication. We hope all the members of the profession may have the benefit of Dr. Cushing's opinions and thoughts, as expressed in the essay.

The regular subject was then introduced for discussion.

### Mechanical Dentistry.

Dr. Cameron, being called upon, said, I will, no doubt, be regarded by many as an old fogy in my ideas, because I have but very few good words to say for the popular vulcanite work. Those who recommend in their offices, vulcanite as being better than gold for plates in all cases, will probably consider me far behind the times; for I cannot agree with them. I always, when asked for my honest opinion in regard to this matter, recommend as above all other materials for artificial work, good gold. I do not advise the use of either silver or vulcanite, though I use both. I do not consider silver suitable for the mouth at all, though in very many cases I advise its use before vulcanite. I tell my patients that gold is the best material I can use—platinum also makes a good base. I tell them gold work is stronger, easier kept clean, more readily repaired, not so thick and "bunglesome" as the vulcanite. When they take the gold they never come back asking me to change it for any other material. This is not always the case when they take the vulcanized rubber.

I have frequently, after making entire sets on rubber, had the patient come back, in a few weeks or months, thoroughly disgusted and wanting a change to gold. For temporary dentures, where the patient can't afford gold, I prefer silver. When I cannot get a rim of vulcanite over the artificial gums, I have several times refused to make work on vulcanite, unless the patient will take the entire risk of a bad fit and frail piece. One argument offered in favor of the vulcanite is that it is cleaner, or more easily kept clean, than metal plates. True there are no interstices for the food, and secretions of the mouth to get into; but the whole plate becomes covered with a slimy deposit of apparently vitiated mucus, which must be exceedingly disagreeable, and salivary calculus seems to have a special affinity for the rims and posterior palitine portions of the rubber plate. In artficial work, I discard clasps entirely and depend upon atmospheric pressure, and as good fits as possible, to keep my plates in their places.

Dr. Rosson asked if Dr. C's sets ever drop down.

Dr. Cameron, oh, yes! have had them drop out of the mouth, and I don't know how many yards away. My reasons for not using clasps is the injury they most certainly cause the teeth to which they are attached. For full impressions I use plaster, and for partial cases, beeswax. I make my casts of zinc and lead, and have but very little trouble with the changing of plates, from the shrinkage of the zinc casts. After having ground and adapted my teeth to the plates, (and rim in entire dentures,) I always try them in the mouth, see them, arrange and fix them just as I expect them to look and fit, before I finish them. From the mouth I take them, and invert in sand and plaster, in about equal parts. I use no stays, or wires, or clamps, to prevent warping, and never calculate on trouble from this quarter, when the work is properly inverted. I make my own solder, taking the gold of the plate to be soldered, and fine sheet brass, in the proportion of two grains of brass to a pennyweight of gold. I melt the gold first, and then introduce the brass, which should be stirred about thoroughly. With this, if the borax and plate are clean, and the whole piece heated to a cherry red color, the soldering is but very little trouble; and we have a smooth easy flowing solder, that will not tarnish in the mouth.

Dr. TALBERT said, I think we have much less trouble with work when we use good gold for our plates. I prefer always to use gold of 20 or 21 carets fine, and believe that this quality of plate is much less liable to spring or warp under heat. I use compound metal dies, and consider them more accurate than zinc and lead, unless we use our zinc but once or twice, and have it pure in quality. Perhaps if all would use a good quality of gold, and have our laboratories fitted better for manipulating metals, the rubber work would not be in such great demand.

Dr. BERRY had, a few years ago, always taken coin gold, and without melting, simply rolled out his plates. He was then sure of the quality of his plates, and saved the extra trouble of melting and refining gold.

The Society then adjourned to meet next year at the time agreed upon.

On account of an unusual amount of miscellaneous business that required the attention of the Society, the subjects for discussion, as appointed or recommended by the executive committee, were rather briefly discussed. I do not, however, pretend that the above is by any means a full report of all that was said on the regular questions; but if I have gleaned enough to interest the readers of the REGISTER for one short hour, I shall be truly thankful. If I have fixed a little of the "floating knowledge" of the profession in a tangible form, or if I have reported clearly some of the sayings of truth and wisdom that were so abundant during this meeting of the Society, I shall be satisfied with my attempt in my new *role* as reporter of so learned a body as the Mississippi Valley Dental Association.

